# Phosphatases maintain low catalytic activity of SGK1: DNA damage resets the balance in favor of phosphorylation

**DOI:** 10.1016/j.jbc.2023.104941

**Published:** 2023-06-19

**Authors:** Wenxue Gu, Hongyan Zheng, Cecilia M. Canessa

**Affiliations:** 1 School of Medicine, Tsinghua University, Beijing, China; 2 Cellular and Molecular Physiology, School of Medicine, Yale University, New Haven, USA

**Keywords:** PP2A, PP5, FAM122A, DNA-PK, genotoxicity, phosphorylation

## Abstract

The serum- and glucocorticoid-induced kinase 1 (SGK1) promotes cell survival under stress conditions and facilitates the emergence of drug resistance in cancer. The underlying mechanisms of these observations are not fully understood. In this study, we found that SGK1 activity is suppressed by the action of the S/T phosphatases PP5 and PP2A, which constantly dephosphorylate SGK1. Using newly developed anti-phospho SGK1 antibodies and inhibitors of phosphatases, we determined that the high degree of dephosphorylation is caused by two factors: the tendency of SGK1 to unfold, which makes it dependent on Hsp90 chaperone complexes composed of four proteins, Hsp90/CDC37/PP5/SGK1, and where the phosphatase PP5 persistently dephosphorylates SGK1 within the complex. SGK1 binding to PP2A regulatory subunits B55γ and B55δ brings PP2A catalytic subunit close to exposed SGK1 phosphoresidues. A further association of phosphorylated pS37-FAM122A—an endogenous inhibitor of PP2A—to the holoenzyme diminishes dephosphorylation of SGK1 mediated by PP2A. Our study also reveals that genotoxic stress can reverse the dominant impact of phosphatases over kinases by activating the DNA-dependent protein kinase, which enhances mTORC2 activity directed to SGK1. Thus, our results provide insight into a molecular pathway that enables SGK1 to gain phosphorylation and catalytic activity and promote cell survival, potentially diminishing the efficacy of cancer treatments. As the DNA damage response operates in many cancer cells and is further induced by chemotherapies, the findings of this study could have significant implications for the development of novel cancer therapies targeting SGK1.

SGK1 is a protein kinase that belongs to the serine/threonine kinase family (1). It shares many similarities with AKT1, including the same upstream activation pathway and consensus phosphorylation sequence of substrates (2, 3, 4). Both kinases are phosphorylated in the hydrophobic motif (HM) by mTORC2 and in the activation loop by PDK1 (2, 3, 5). Additionally, they enhance growth, stress survival, and promote the emergence of drug resistance in cancer treatments (6, 7, 8). However, the role of AKT1 in these processes is well understood, while much less is known about SGK1. This is because insight into its function and regulation is still in its infancy. More research is needed to understand the specific role of SGK1 in various cellular processes and its potential as a therapeutic target for diseases like cancer.

Despite their similarities, deleting or overexpressing AKT or SGK1 in mice leads to distinct phenotypes, highlighting fundamental differences between the two kinases. Abrogation of AKT1 primarily induces metabolic, growth, and proliferation effects (9, 10), while global deletion of SGK1 only mildly impairs renal sodium and potassium secretion (11, 12). This impairment is attributed to altered expression of ion channels, which are presumably mediated by SGK1 phosphorylation of the ubiquitin ligase Nedd4L (13). Some processes have been assigned exclusively to SGK1, such as the expression and stabilization of the phenotype of TH17 cells (interleukin-17–producing helper T cells) (14) and the magnification of the response to transforming growth factor β by preventing degradation of the transcriptional factors Smad2/3 (15). SGK1 also differs from AKT1 on extensive transcriptional regulation, which can be triggered by a wide range of stimuli, including serum, glucocorticoids, as well as many other hormones and environmental stress factors (16).

High levels of SGK1 expression have been observed in many cancers, which are attributed to various factors, such as promoter methylation and aberrant splicing of mRNA (17, 18). This overexpression of SGK1 often correlates with low or no response to AKT1 inhibitors (19) and other chemotherapeutics (17, 20). However, despite this correlation, few studies have examined the state of SGK1 phosphorylation, which is the most direct indicator of activity, possibly due to assumption that SGK1 phosphorylation state and activity fluctuate in parallel to those of AKT1, as well as limited availability of specific anti-phospho SGK1 antibodies. Nonetheless, this study found that SGK1 exhibits very low phosphorylation levels in resting cells and even upon growth factor stimulation. However, SGK1 phosphorylation markedly increases under genotoxic stress, indicating a potential role in promoting survival and resistance to some chemotherapeutics, particularly AKT1 inhibitors in cancer cells. Therefore, understanding the mechanisms underlying SGK1 activation and its role in promoting cancer cell survival and resistance may provide valuable insights for developing effective cancer therapies.

## Results

### Phosphatases maintain low level of SGK1 phosphorylation in resting and growth factor–stimulated cells

SGK1 exhibits catalytic activity upon undergoing two sequential phosphorylation events. The first event involves the phosphorylation of the carboxyterminal HM at residue S422 by mTORC2, creating a docking site for PDK1 (3). The second event is the phosphorylation of the activation loop at T256 by PDK1 (2). Phosphorylation of the two residues is necessary to activate SGK1. Therefore, the presence of pT256 or pS422 indicates the active state of SGK1. In contrast, AKT activation only requires phosphorylation of the activation loop at T308, while phosphorylation of the HM at S473 by mTORC2 maximally enhances AKT1 catalytic activity (4).

**Figure 1:**
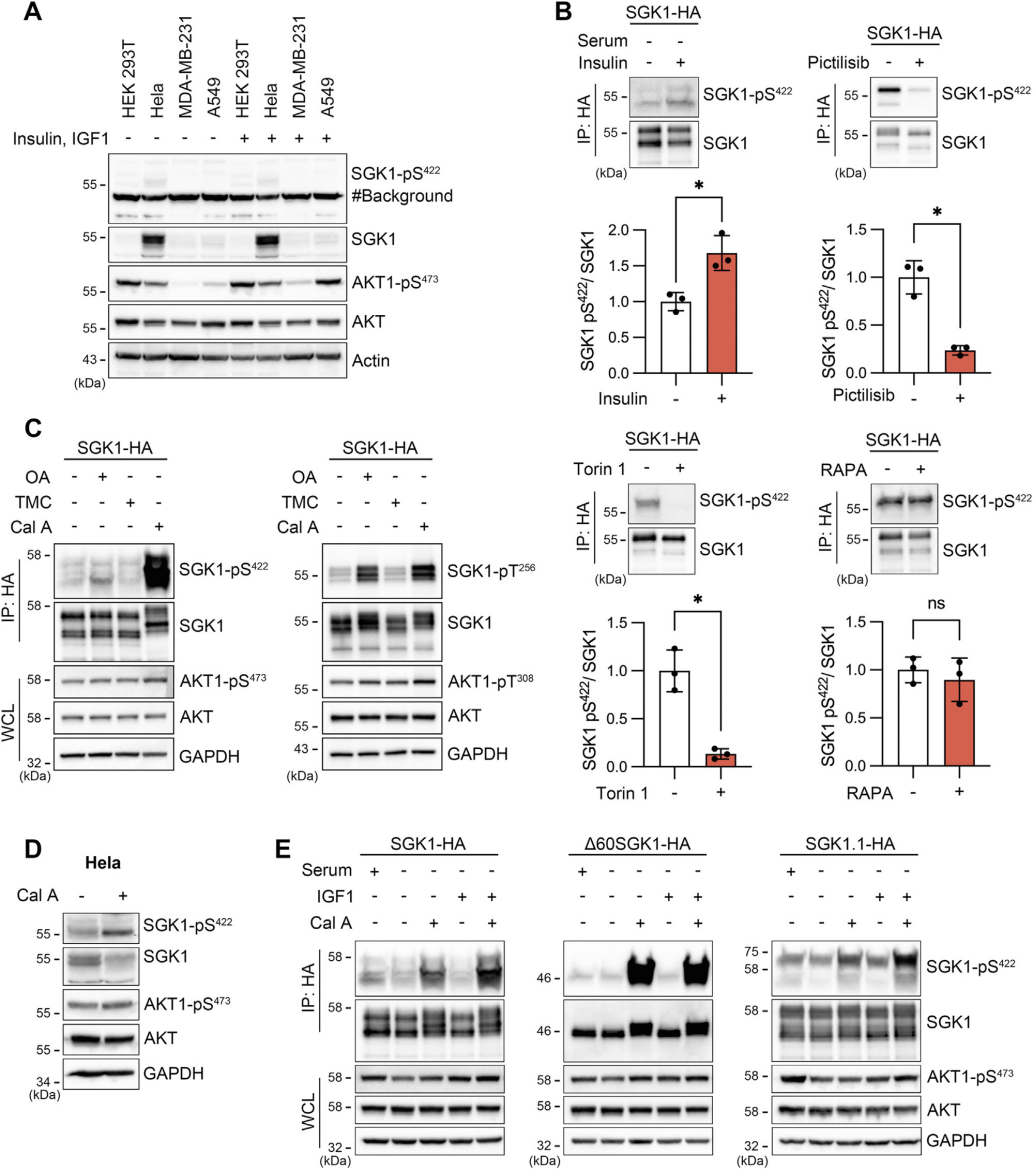
**Phosphatases maintain low level of SGK1 phosphorylation in resting and growth factor–stimulated cells.***A*, SGK1 expression and phosphorylation levels in resting or 30 min treatment with growth factors (20 μg/ml insulin and 250 ng/ml IGF1) in the indicated human cell lines. Upper two rows are western blots of pS422 and total SGK1. The strong ∼45 kDa band produced by the commercial anti-pS422-SGK1 antibody marked by # is background. Lower rows show pS473 AKT1 and total AKT from the same samples and actin as loading control. *B*, changes in pS422-SGK1 induced by insulin alone in serum-starved cells and with inhibitors of PI3K (1 μM Pictilisib for 2 h), mTORC1-2 (200 nM Torin 1 for 30 min), and mTORC1 (20 μg/ml rapamycin for 30 min). Quantification of relative SGK1 phosphorylation. Data are mean ± SD from three independent experiments. Insulin, Torin 1, Pictilisib, Rapa ∗*p* < 0.03 by Welch’s *t* test. *C*, phosphatase inhibitors effect on pS422- and pT256-SGK1, and pS473- and pT308-AKT1. *D*, Cal A effect on endogenous pS422-SGK1 and pS473-AKT1 in HeLa. *E*, phosphorylation changes of the corresponding pS422 in three isoforms of SGK1 induced by serum, IGF1, or Cal A. Levels of pS473-AKT1 induced by the same treatments are shown for comparison. Cal A, Calyculin A; OA, okadaic acid; SGK1, serum- and glucocorticoid-induced kinase 1; TMC, tautomycetin.

**Figure S1:**
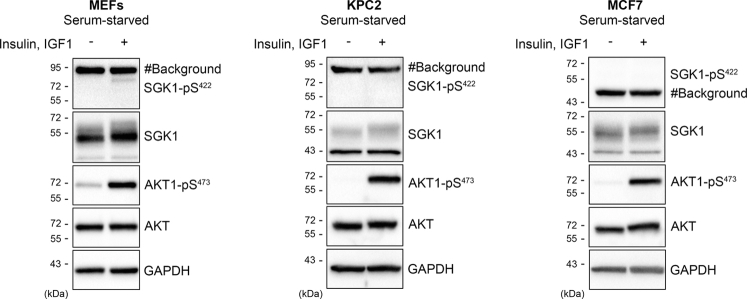
**Endogenous SGK1 phosphorylation in resting and stimulated MEFs and cancer cell lines.** The indicated cells were serum-starved for 20 h before stimulation with both insulin and IGF1 (20 μg/ml insulin and 250 ng/ml IGF1). MEFs were derived from transgenic mice expressing SGK1-3xFlag (25) and blotted with SGK1 antibody. For detection of phosphorylated SGK1 in MEFs and KPC2 cells, membranes were blotted with our raised anti-pS422 SGK1 antibody, whereas MCF7 was blotted with a commercial antibody (Invitrogen #44-1264G). MEF, mouse embryonic fibroblasts; KPC2, mouse pancreatic ductal adenocarcinoma; MCF7, human breast cancer epithelial line.

A commercial human SGK1 anti-pS422 antibody was employed to detect HM phosphorylation of SGK1 in cell lysate of 293T and the indicated human cancer cell lines. However, the phosphorylation of SGK1’s HM was found to be very low or undetectable even after treatment with insulin and insulin-growth factor (IGF1) (Figs. 1*A* and S1). This observation may be attributed, in part, to the low expression of SGK1 in most cell lines. Even in HeLa cells, which exhibited the highest level of endogenous SGK1 expression among the examined cells, the HM phosphorylation was minimal. In contrast, the basal phosphorylation of AKT1’s HM was readily detected in the same cells, although the magnitude of insulin-induced phosphorylation varied considerably among the different lines, likely reflecting variations in endogenous PI3K activity.

**Figure S2:**
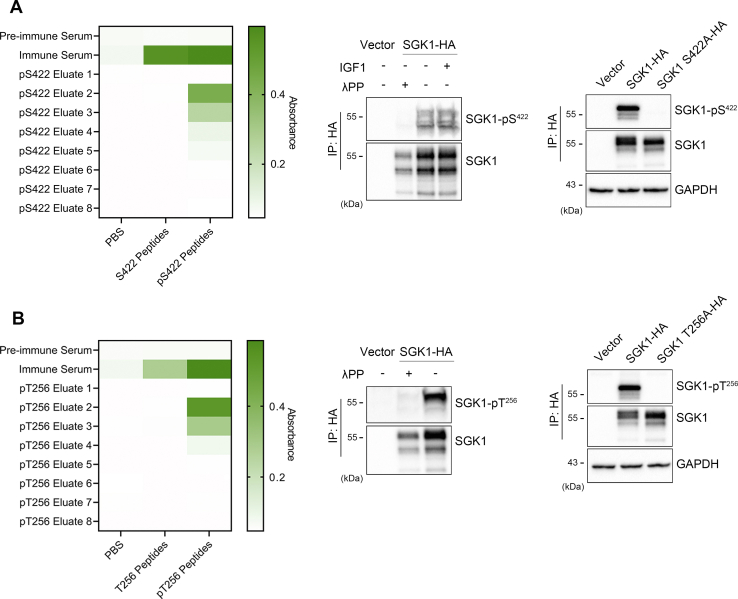
**Purification and validation of rabbit pS422 and pT256 antibodies**. *A*, rabbit sera were first affinity purified with a S422-peptide column and then with a pS422-peptide column. Intensity of ELISA signals generated by eight consecutive elution fractions from the pS422-peptide affinity column with 0.1 M glycine, pH 2.5 and tested against PBS, S422 peptide, or pS422 peptide as substrates. The *green* gradient indicates values of absorbance. *B*, reactivity of purified anti-pS422 antibodies tested by Western blots of lysates from 293T transfected with empty vector or SGK1 WT treated with lambda phosphatase (lPP) or IGF. *C*, reactivity of purified anti-pS422 antibodies tested with SGK1 WT and mutant S422A. *D*, analysis of rabbit sera affinity purified with a pS256-peptide column tested by ELISA as in (A). *E*, reactivity of anti-T256 antibodies on cell lysates treated with lPP. *F*, reactivity of anti-T256 antibodies on lysates from cells transfected with SGK1 WT and mutant T256A.

To investigate further the regulation of SGK1 phosphorylation, rabbit polyclonal anti-phospho-antibodies specific for pS422 and pT256 were developed (Fig. S2). The presence of transfected SGK1-HA was easily detected in western blots. However, neither the commercial nor the newly developed anti-phospho-antibodies detected the phosphorylated protein by directly blotting 293T cell lysates. The detection required enrichment through immunoprecipitation of SGK1 followed by blotting with anti-pS422 or anti-pT256 antibodies, a procedure employed in all experiments in this study. SGK1 phosphorylation increased slightly with insulin and decreased significantly by inhibiting of PI3K (Pictilisib) or mTORC1/2 (Torin 1), while inhibition of only mTORC1 (Rapamycin) had no effect (Fig. 1*B*). These results suggest that the observed phosphosignal aligns with the established upstream activation pathway of SGK1 (2, 3). It is noteworthy that the multiple bands observed in SGK1 blots are caused by N-terminal truncations of SGK1, which are generated by alternative initiation of translation rather than phosphorylation (21).

The modest phosphorylation of SGK1 could be due to the low affinity of mTORC2 towards the S422 site of SGK1 or the presence of phosphatases that preferentially target SGK1. To explore this possibility, 293T cells expressing SGK1 were treated with inhibitors of the most abundant cellular phosphatases: PP1, PP2A, and PP5 (22, 23, 24, 25). Okadaic acid and, to a large extent, Calyculin-A significantly increased pS422- and pT256-SGK1 signals, while their impact on the corresponding AKT1 residues, pS473 and pT308, was minimal (Fig. 1*C*). Calyculin-A also elevated the phosphorylation of endogenous SGK1 in HeLa cells and all three SGK1 isoforms: canonical, Δ60SGK1, and SGK1.1 (Fig. 1, *D* and *E*). The isoforms differ in their amino termini but possess identical catalytic domains and HMs (26, 27).

These findings collectively suggest that the low phosphorylation state of SGK1 in resting and growth factor–stimulated cells is due to a dominant activity of phosphatases over kinases. This implies that SGK1 regulation differs significantly from that of AKT1, which maintains strong phosphorylation even in nonstimulated cells, such as 293T and HeLa.

### Unstable SGK1 structure exposes phosphorylated residues to phosphatases

**Figure 2:**
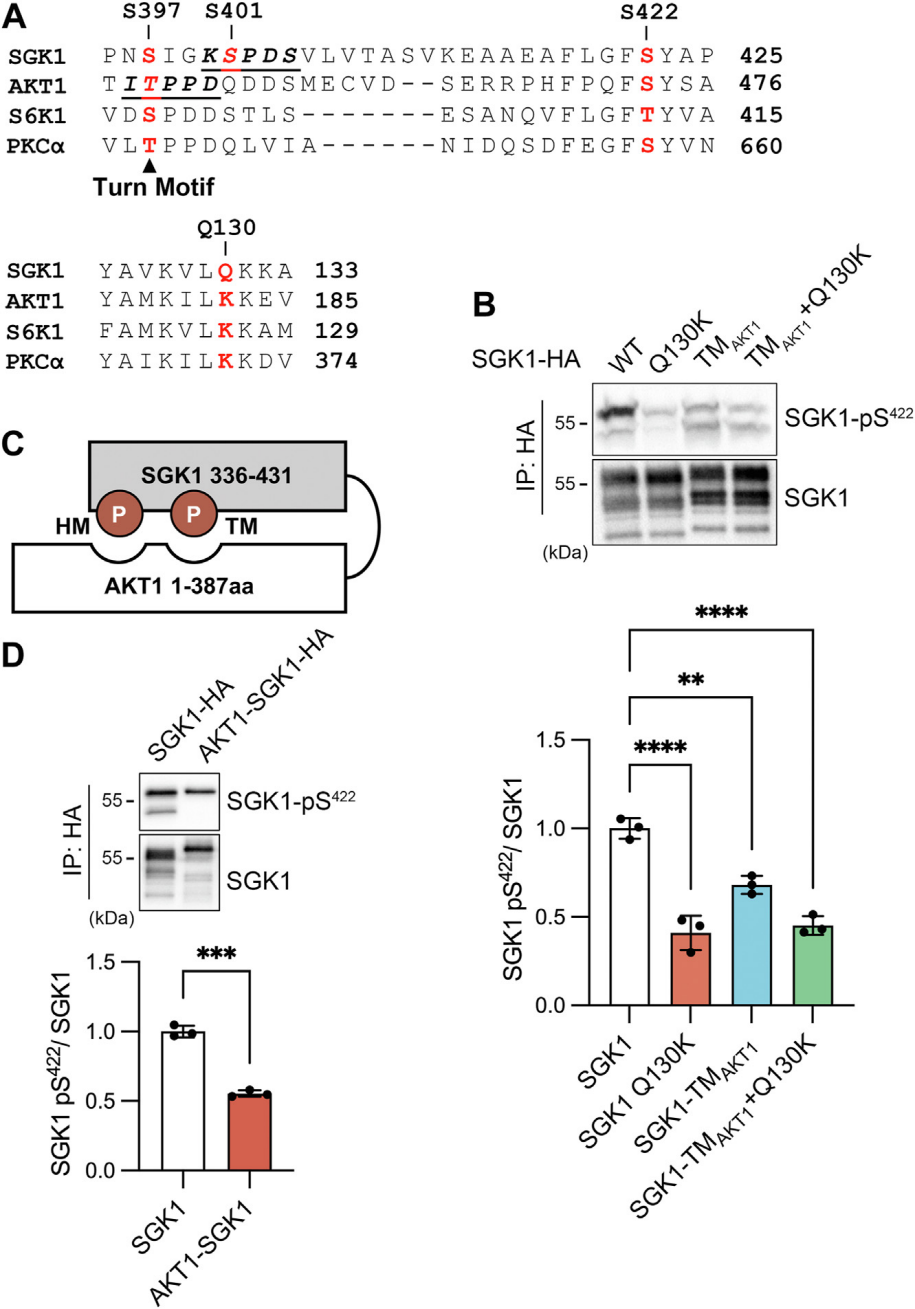
**The putative TM-binding pocket does not protect dephosphorylation of S422.***A*, sequence alignment of C termini of indicated kinases. Proven turn motif residues known to be phosphorylated in other AGC kinases are indicated in *red*. Below is the sequence alignment of the positively charged pocket that binds the phosphorylated turn motif. *B*, Western blot of pS422 in SGK1 WT, single mutant Q130K, replacement of turn motif for that of AKT1 (TM_AKT1_), and both Q130K and TM_AKT1_. Analysis of the mean ± SD of n = 3 experiments by one-way ANOVA and Dunnett’s post hoc test: ∗∗*p* = 0.0011, ∗∗∗∗ *p* < 0.0001. *C*, cartoon representation of AKT1-SGK1 chimera where phosphorylated residues of the TM and HM dock on distinct sites located in the N-lobe of the kinase domain. Numbers indicate the amino acids corresponding to each kinase. *D*, Western blot of pS422 in SGK1 WT and AKT1-SGK1 chimera. Quantification analysis: bars are the mean ± SD of n = 3 independent experiments. Statistical difference examined by Welch’s *t* test: ∗∗∗*p* = 0.0004. SGK1, serum- and glucocorticoid-induced kinase 1.

It has been suggested that mTORC2 plays a role in phosphorylating the turn motif (TM) of AKT at T450 during protein translation (28). This phosphorylation allows the TM to bind to a pocket rich in basic residues located in the N-terminal lobe of the catalytic domain. The binding occurs in a zipper-like fashion as it brings the phosphorylated S473 residue in the HM to its own binding site, which is also located in the N-terminal lobe (29). This mechanism effectively shields the two phosphorylated residues, preventing them from being removed (Fig. 2*C*).

**Figure S3:**
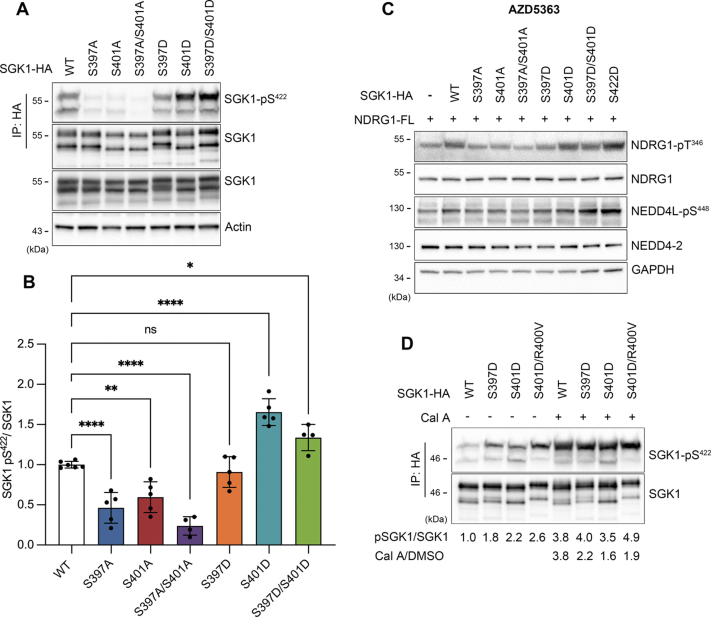
**Mutations of S401 in SGK1 turn motif impact the phosphorylation level at pS422.** *A*, S422 phosphorylation level of SGK1 WT and indicated mutants in transfected 293T cells. *B*, quantification analysis of pS422 signal as the ratio of pS422/total SGK1. Bars indicate the mean ± SD of four to five independent experiments. Statistical analysis by one-way ANOVA and Dunnett’s post hoc test: ∗*p* < 0.02, ∗∗*p* < 0.002, ∗∗∗∗*p* < 0.0001. *C*, WB of NDRG1 pT346 and NEDD4L pS448 (two substrates of SGK1) phosphorylation level with indicated SGK1 constructs and the presence of AKT1 specific inhibitor AZD5363 (10 µM) applied for 4 h. The inhibitor was added to identify the component of NDRG1 phosphorylation mediated by SGK1 as the site T346 is also phosphorylated by AKT1. *D*, WB of pS422 and total SGK1 of WT SGK1 and indicated mutants ± 100 nM Cal A for 15 min.

It remains unclear whether the turn motif and hydrophobic pocket of SGK1 function similarly to those of AKT1. SGK1 has two potential phosphorylation sites in its carboxy terminus TM, namely S397 and S401 (Fig. 2*A*). To investigate this, alanine or aspartic acid substitutions were made to prevent or mimic phosphorylation, respectively. The alanine substitutions decreased the pS422 signal, while the S401D mutant significantly increased it (Fig. S3, *A* and *B*). Additionally, phosphorylation of the TM and HM increased SGK1’s activity, as evidenced by increased phosphorylation of two known substrates, NDRG1 (30) and NEDD4L (15) (Fig. S3*C*). However, phosphorylation of the TM only partially protected SGK1 from phosphatases, as demonstrated by the persistent and significant effect of Calyculin A on the mutants (Fig. S3*D*).

In SGK1, residue Q130 replaces a conserved lysine found in the phosphorylated TM-binding pocket of other similar kinases, potentially reducing its affinity to phosphorylated TM (29) (Fig. 2*A*). Surprisingly, the substitution of Q130 with lysine in SGK1, as well as the replacement of the turn motif with that of AKT1 (TM_AKT1_), did not prevent dephosphorylation of S422; in fact, it was even more severe in these mutants (Fig. 2*B*). Furthermore, a chimera consisting of the AKT1 catalytic domain and the SGK1 carboxy terminus also showed a low level of pS422 (Fig. 2, *C* and *D*). These results suggest that the phosphorylation of TM and its putative binding site in the catalytic domain do not impede phosphatase access to SGK1’s critical phosphoresidues.

### PP5 dephosphorylates SGK1 in complex with Hsp90 and CDC37

**Figure 3:**
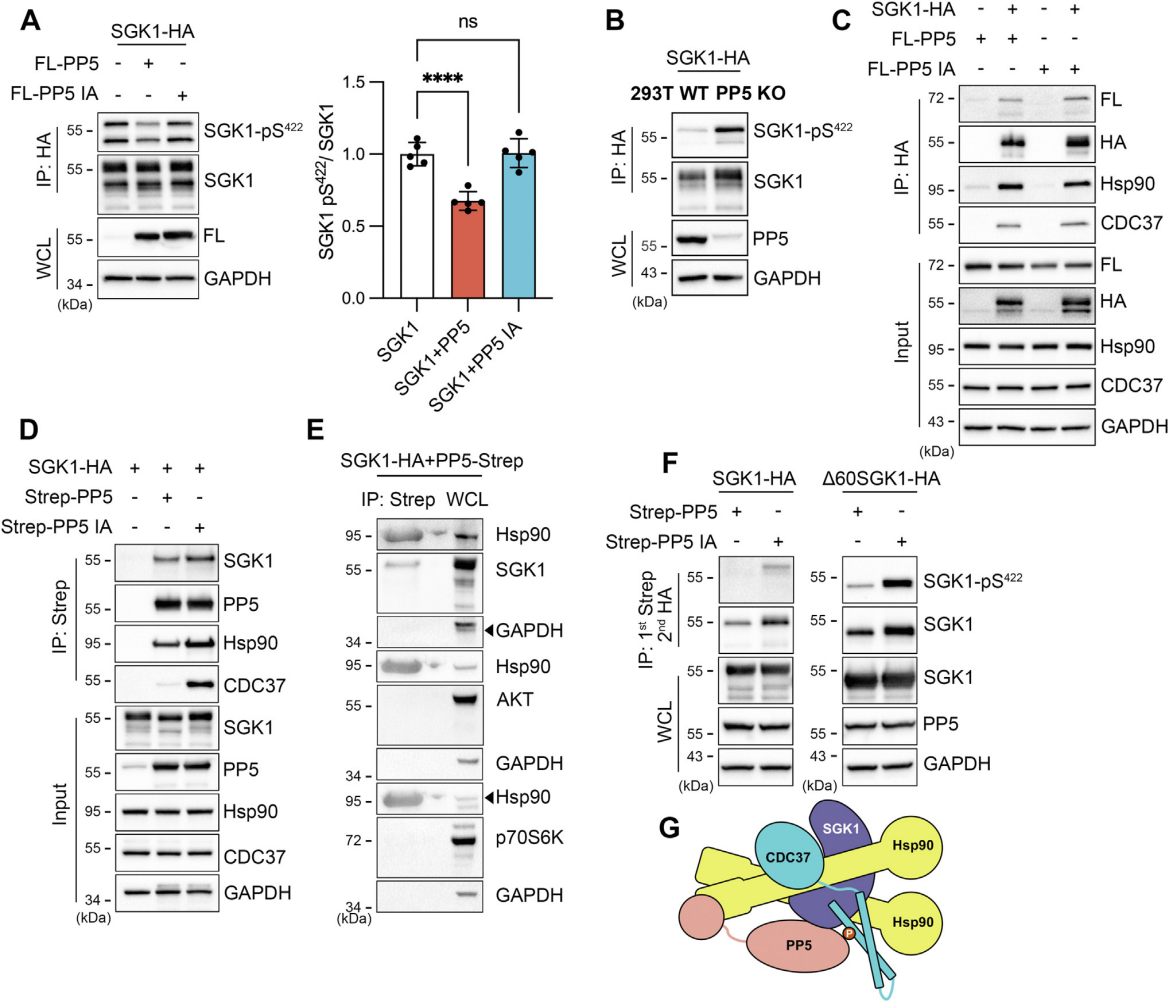
**SGK1 associates with Hsp90/CDC37/PP5 complex where it is dephosphorylated.***A*, pS422-SGK1 in 293T expressing WT or catalytically inactive PP5 (PP5-IA, N303A/H304A). Statistical analysis of the mean ± SD of five independent experiments by one-way ANOVA and Dunnett’s post hoc test: ∗∗∗∗*p* < 0.0001. *B*, pS422-SGK1 in WT and PP5 KO 293T cells. *C*, pulldown of SGK1-HA with anti-HA magnetic beads reveals the presence of SGK1, PP5, CDC37, and Hsp90. *D*, pulldown of active or inactive PP5 with Strep-Tactin coprecipitates SGK1, endogenous Hsp90, and CDC37. *E*, Hsp90, SGK1, AKT1, and p70S6K coprecipitated with PP5 (pulldown by Strep-Tactin resin) compared to the abundance of these kinases in the whole cell lysate (WCL). *F*, SGK1-HA and Δ60SGK1-HA pull-down by Strep-PP5 WT or inactive and analyzed with anti-phospho-S422 antibody. *G*, cartoon representation of the hypothetical SGK1/Hsp90/CDC37/PP5 complex. SGK1, serum- and glucocorticoid-induced kinase 1.

In a previous study, we demonstrated that the stability of SGK1 is heavily dependent on the chaperone proteins Hsp90 and CDC37 (31). Building on that research and the observation that calyculin-A increases SGK1 phosphorylation (Fig. 1, *C* and *D*), it was hypothesized that PP5 might dephosphorylate SGK1 while associated with the Hsp90/CDC37/PP5 complex. The N-terminal domain of PP5 contains three consecutive tetratricopeptide repeats that bind to the Hsp90 C-terminus tetratricopeptide repeat–binding site (EEVD) and activate PP5 (32). Here we found that expression of WT PP5 reduced pS422-SGK1 levels, whereas a catalytically inactive PP5 (PP5 IA: N303A/H304A) (33, 34) did not have this effect (Fig. 3*A*), and knockout of PP5 in 293T increased pS422 level above that of WT cells, consistent with SGK1 being a substrate of PP5 (Fig. 3*B*). To investigate the compartment where PP5 dephosphorylates SGK1, 293T cells transfected with SGK1-HA and FL-PP5 or SGK1-HA and Strep-PP5 (active or inactive) were used for pulldowns. All four proteins of the complex (SGK1, PP5, Hsp90, and CDC37) were detected regardless of whether the pulldown was conducted with SGK1 or PP5 (Fig. 3, *C* and *D*), and SGK1 was found to be dephosphorylated when PP5 was active within the chaperone complex but not with the inactive PP5 mutant (Fig. 3*F*). These findings indicate that PP5 dephosphorylates SGK1 in addition to CDC37, a known PP5 substrate (35).

Complexes exclusively containing inactive PP5 displayed enhanced associations with all proteins within the complex, particularly CDC37 (Fig. 3*D*). This observation suggests that the dephosphorylation of CDC37 by PP5 facilitates its dissociation from the chaperone complex and promotes the disassembly of other components. However, when both WT PP5 and PP5-IA were present in the complexes, this effect was less pronounced (Fig. 3*C*). Importantly, related kinases such as AKT1 and p70S6K were not detected in the pulldowns (Fig. 3*E*), likely due to their higher structural stability than SGK1, which reduces their reliance on chaperones. Taken together, these findings indicate that the high propensity of SGK1 to unfold makes it dependent on the Hsp90/CDC37 complex, where PP5, one of its components, dephosphorylates SGK1 to maintain a low phosphorylated state.

### SGK1 interacts with PP2A containing B55γ/δ and B56β subunits

**Figure 4:**
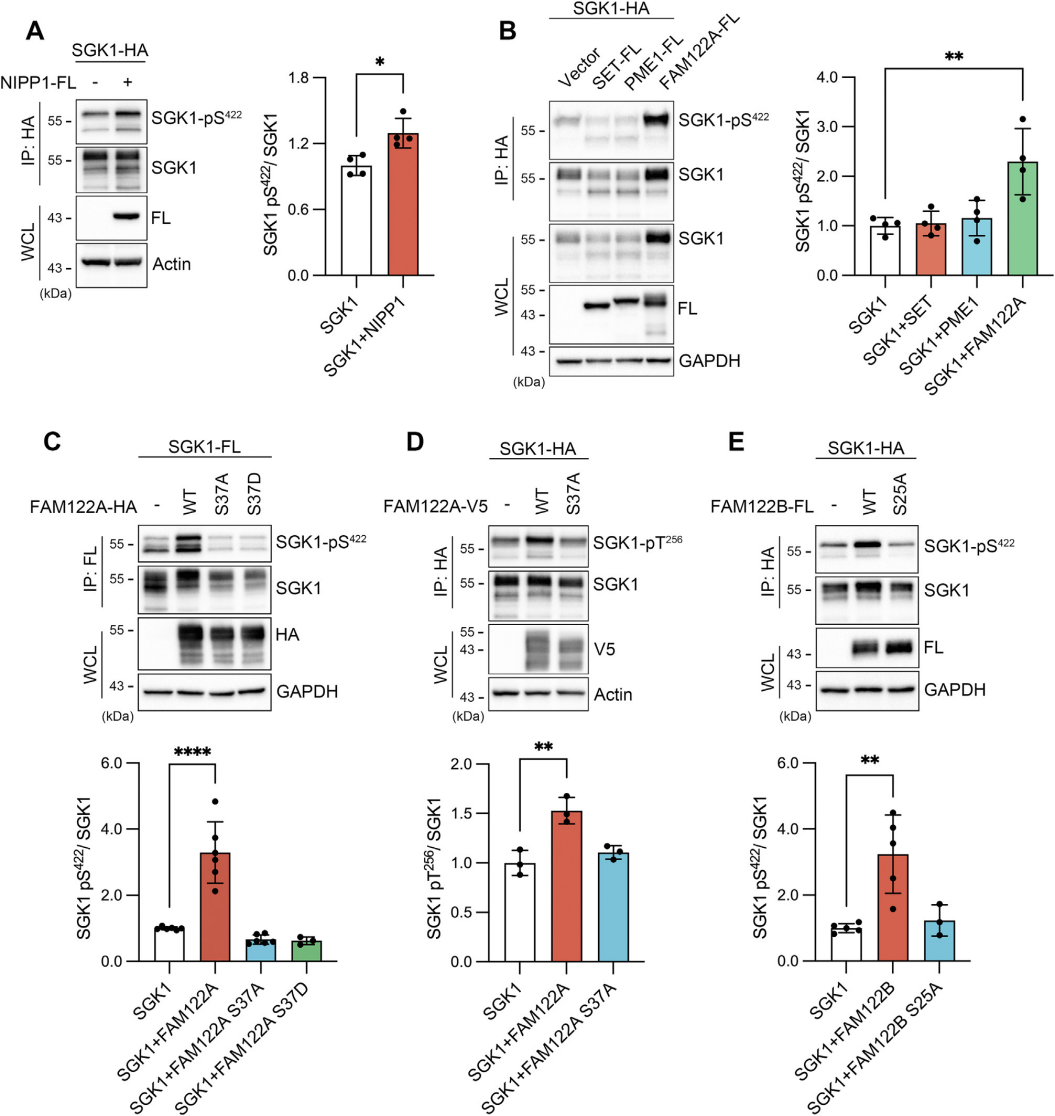
**Impact of endogenous phosphatase inhibitors on SGK1 phosphorylation.***A*, effect of NIPP1, an endogenous inhibitor of PP1 on the level of pS422. Quantification analysis: bars are the mean ± SD of n = 4 independent experiments. Statistical difference examined by Welch’s *t* test: ∗*p* = 0.0138. *B*, effect of endogenous PP2A inhibitors on S422 phosphorylation. Quantification of four independent experiments shown as mean ± SD, ∗∗*p* = 0.002. *C* and *D*, impact of FAM122A WT and S37A/D mutants on S422 and T256 phosphorylation of SGK1. Quantification of three to six independent experiments shown as mean ± SD, ∗∗*p* = 0.0029, ∗∗∗∗*p* < 0.0001 by one-way ANOVA and Dunnett’s post hoc test. *E*, impact of FAM122B WT and S25A mutant on S422 phosphorylation. Quantification of three to five independent experiments shown as mean ± SD, ∗∗*p* = 0.0024 by one-way ANOVA and Dunnett’s post hoc test. SGK1, serum- and glucocorticoid-induced kinase 1.

**Figure S4:**
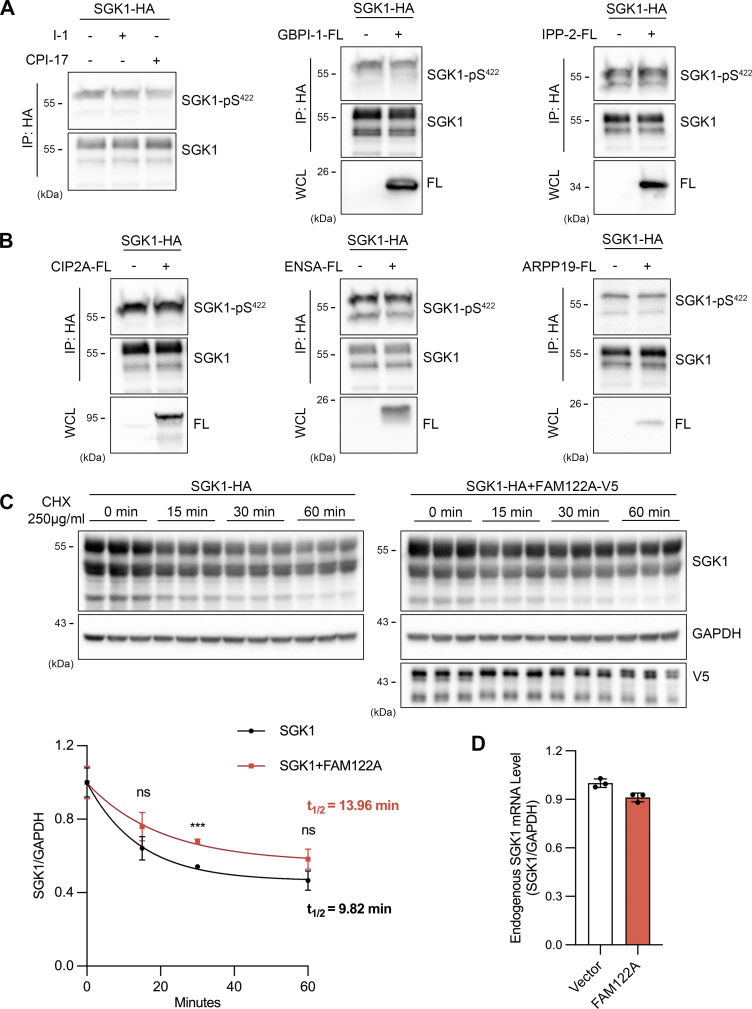
**Impact of endogenous phosphatase inhibitors on SGK1 phosphorylation, protein stabilization, and transcription.** *A* and *B*, WB of pS422-SGK1 intensity in the presence of the indicated endogenous inhibitors of PP1 and PP2A, respectively. *C*, stability of SGK1 measured by cycloheximide (CHX) chase ± FAM122A. The graph shows the mean intensity of three replicas for each data point ± SD. Lines are the fit to a single exponential. Calculated *t_1/2_* are indicated. Statistical analysis unpaired t-test, ∗∗∗*p* = 0.0003. *D*, qPCR analysis of endogenous SGK1 in 293T empty vector–transfected control and expressing FAM122A.

The impact of PP1 and PP2A on the state of SGK1 phosphorylation was examined by expressing the corresponding endogenous inhibitors of these phosphatases. Among the PP1 inhibitors tested (inhibitor-1/PPR1A, inhibitor-2/IPP-2, CPI-17/PP14A, GBPI/PP14D, NIPP1) (36, 37), only NIPP-1 slightly increased SGK1 phosphorylation (Figs. 4*A* and S4*A*); whereas among the PP2A inhibitors (SET, PME-1, FAM122A, CIP2A, ENSA, ARPP-16/19), only FAM122A (38, 39, 40, 41) significantly increased phosphorylation (2.5-fold), indicating that SGK1 is also a target of PP2A (Fig. 4*B* and S4, *B*–*D*).

**Figure S5:**
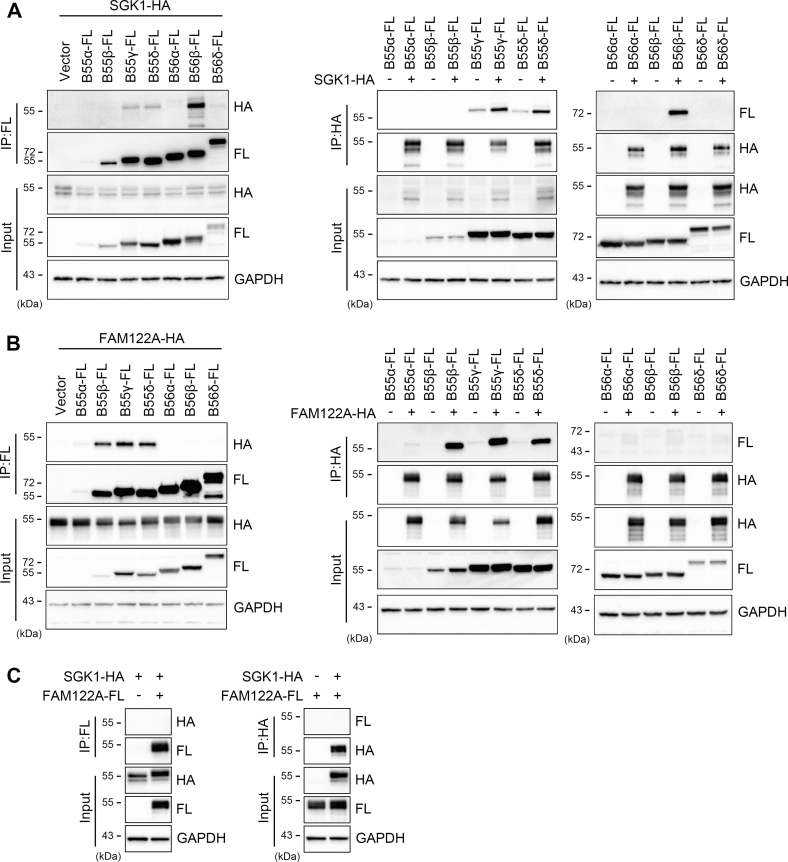
**Association of SGK1 and FAM122A to B subunits of PP2A.** *A*, Western blots of SGK1-HA pull-downs of the indicated B55 or B56 subunits of PP2A, and reverse experiments of B55-FL or B56-FL pull-downs of SGK1-HA. *B*, Western blots of pull-downs of FAM122A-HA with B55-FL or B56-FL subunits and reverse experiment of pull-downs of B55-FL or B56-FL subunits with FAM122A-FL. *C*, Western blots of pull-downs with HA or FL show no interactions of SGK1-HA and FAM122A-FL.

PP2A is made of three subunits: catalytic C, scaffolding A, and various regulatory B subunits that confer substrate specificity to the complex (40). We first looked for physical interactions between B subunits of PP2A and SGK1. An interaction was considered significant only when the co-IP was positive in both directions. With these criteria, associations of SGK1 were detected with B55γ, B55δ, and most strongly with B56β (Fig. S5*A*). Owing to the low expression of B55α, we could not confirm interaction of SGK1 with this subunit. We also examined whether FAM122A binds to B subunits. Co-IP assays detected robust signals of FAM122A with B55β, B55γ, and B55δ; low level of B55α expression again prevented a definitive conclusion with this subunit. None of the B56 subunits associated with FAM122A (Fig. S5*B*). On the other hand, direct association of SGK1 and FAM122A was not found (Fig. S5*C*). These results support a model wherein PP2A holoenzymes containing B55γ/δ subunits associate to and dephosphorylate SGK1, and binding of FAM122A to these complexes inhibits PP2A activity thereby increasing SGK1 phosphorylation.

### Phosphorylation of S37- FAM122A is required to modulate SGK1

It has been recently reported that the checkpoint kinase 1 (Chk1) phosphorylates FAM122A at residue S37 creating a 14-3-3 binding site that leads to its translocation from nucleus to cytosol (42). We asked whether pS37 in FAM122A is also required to increase the phosphorylation state of SGK1. The substitutions S37A and S37D/E in FAM122A eliminated the increase of pS422- and pT256-SGK1 (Fig. 4, *C* and *D*). We also found that a different isoform, FAM122B (61.5% identical to FAM122A encoded by an independent gene: GeneID:159,090), also increased SGK1 phosphorylation, and the corresponding mutation S25 abolished the effect (Fig. 4*E*).

**Figure 5:**
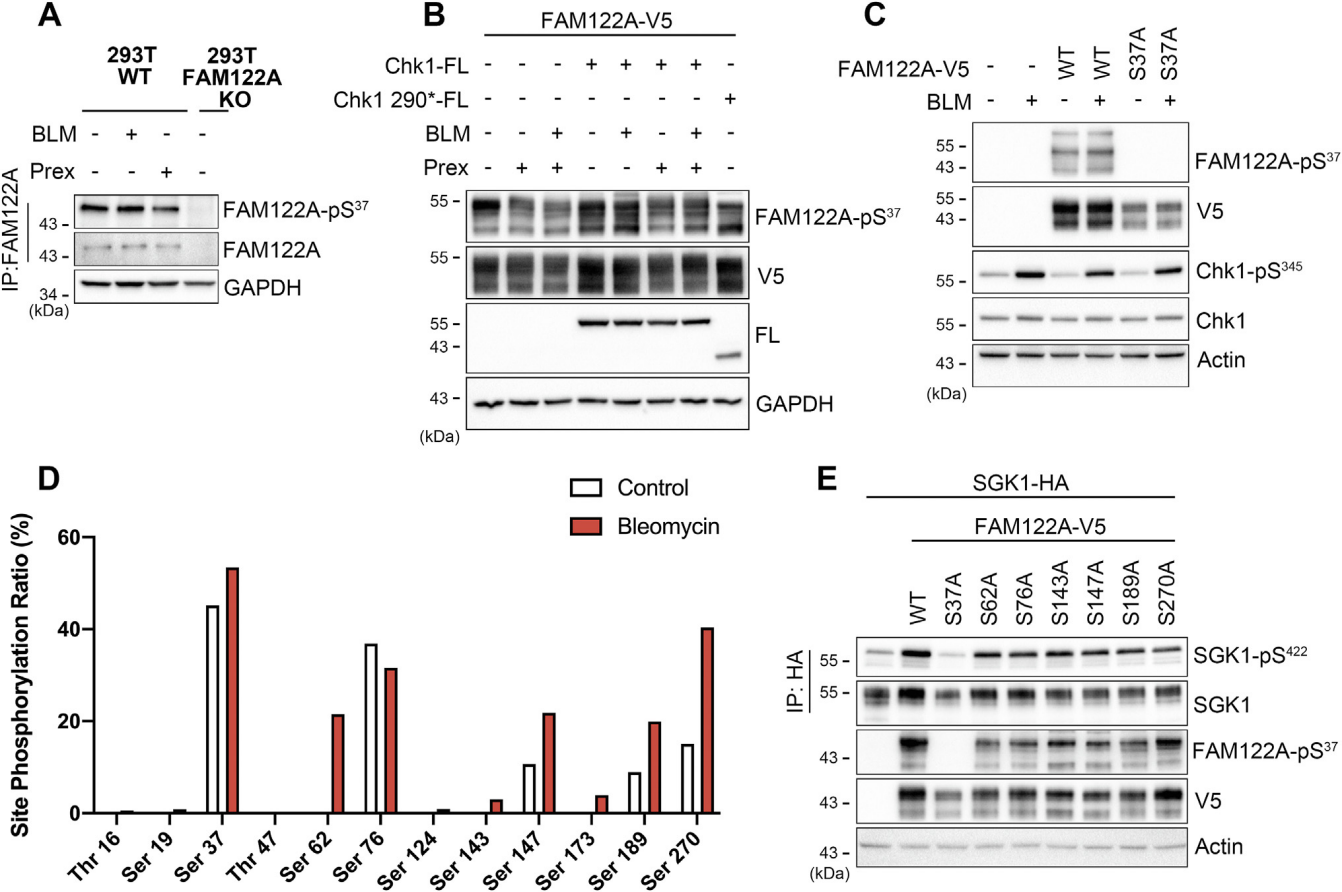
**Phosphorylated sites in FAM122A and their effects on pS422 of SGK1.***A*, phosphorylation level of endogenous pS37 of FAM122A induced by DNA breaks (30 μg/ml bleomycin for 6 h) or inhibition of Chk1 (100 nM prexasertib for 6 h) in 293T WT and FAM122A KO cells. *B*, phosphorylation level of FAM122A pS37 from 293T transfected with FAM122A and Chk1-FL, a constitutive active truncated form Chk1 290∗-FL, treatment with BLM or prexasertib (Prex). *C*, phosphorylation level of pS37 from 293T transfected with FAM122A or S37A mutant treatment with BLM or DMSO control. Endogenous total Chk1 and phosphorylated Chk1 (pS345) are indicated for each condition. *D*, MS phosphopeptide analysis of FAM122A in 293T treated with 30 μg/ml bleomycin or DMSO control for 6 h. *E*, impact of neutralizing phosphorylation of individual FAM122A serine residues on pS422-SGK1 levels. BLM, bleomycin; Chk1, checkpoint kinase 1; SGK1, serum- and glucocorticoid-induced kinase 1.

Given the functional importance of pS37, we raised and affinity purified a FAM122A anti-pS37 rabbit serum to examine the phosphorylation state of FAM122A. 293T or FAM122A KO cells were treated with bleomycin (BLM) to induce DNA breaks and activation of Chk1 or with an inhibitor of Chk1 (prexasertib). Unexpectedly, none of these treatments changed the state of p37-FAM122A phosphorylation (Fig. 5, *A* and *B*), even though endogenous Chk1 was active as indicated by its phosphorylation at residue pS345 (43) (Fig. 5*C*).

These results were additionally supported by mass spectrometry analysis of FAM122A phosphopeptides conducted in control and BLM-treated cells. Figure 5*D* shows that FAM122A is phosphorylated in multiple sites, mainly S37 and S76, and BLM does not alter the pattern of S37 phosphorylation. Substitution of each of the phosphorylated sites by alanine did not change pS422-SGK1 or pS37-FAM122A levels, only pS37 was sufficient and necessary to induce these effects (Fig. 5*E*), indicating it is the only phosphorylated residue with functional impact on SGK1, and pS37 is independent of Chk1 activity. Furthermore, an *in vitro* assay that incorporates radioactive phosphate into the FAM122A peptide containing S37 did not detect phosphorylation by 245 S/T protein kinases that included Chk1/2 (KinaseFinder by Reaction Biology) (Table S1). Overall, these results suggest that phosphorylation of FAM122A at residue S37 by an unknown kinase, rather than Chk1, is necessary and sufficient for the regulation of SGK1 phosphorylation and that FAM122B may play a similar role through phosphorylation of residue S25.

### The DNA damage response signals through DNA-PK to increase phosphorylation of SGK1 and cell survival

**Figure 6:**
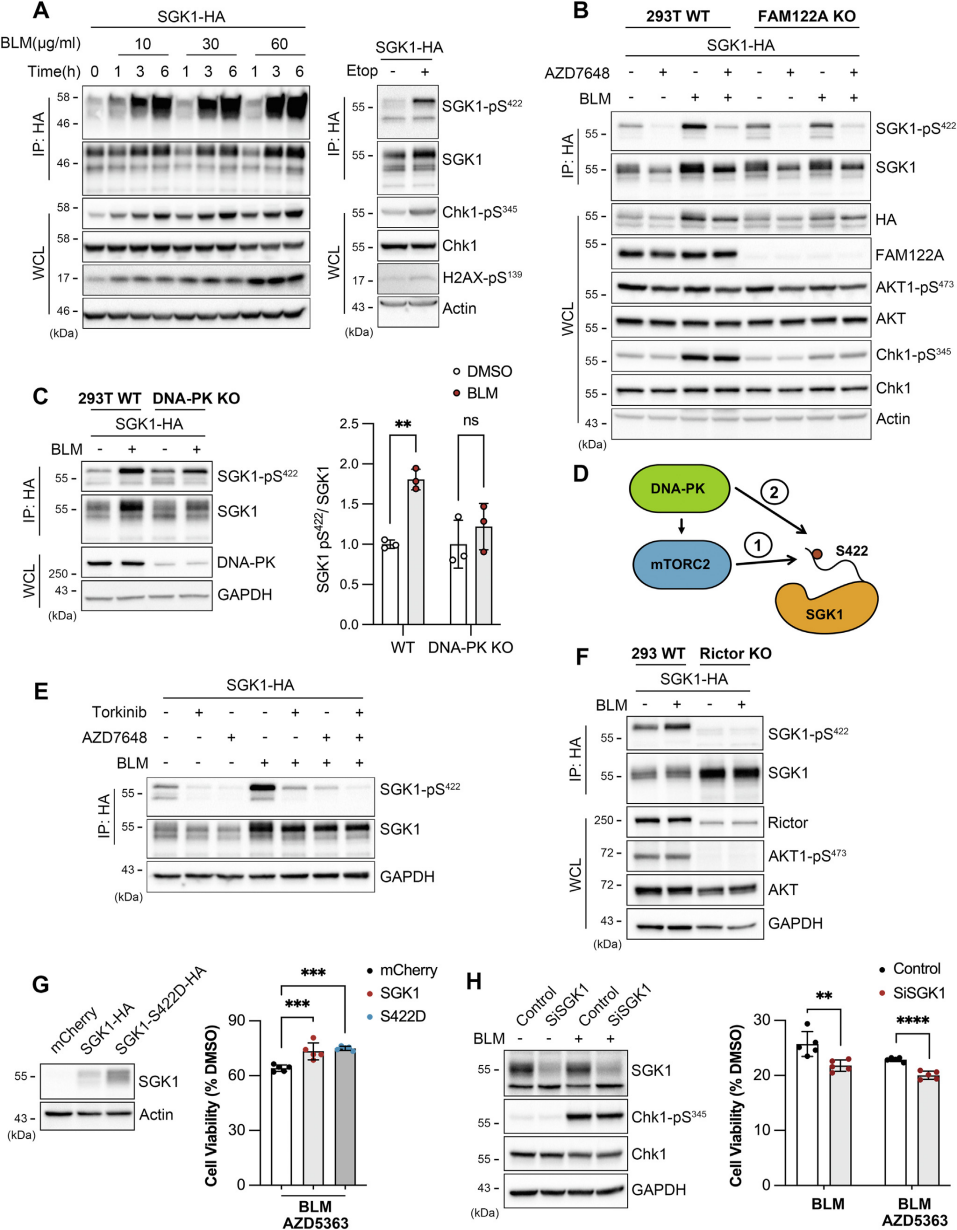
**Genotoxic stress induces phosphorylation of SGK1 mediated by DNA-PK.***A*, time- and dose-dependent increases of pS422-SGK1, pS345-Chk1, and γH2AX (pS139-H2AX) mediated by BLM and etoposide (1 μM for 24 h). *B*, impact of DNA-PK inhibition (1 μM AZD7648 for 6 h) on pS422-SGK1, pS473-AKT1, p345-Chk1 in control and BLM- (30 μg/ml for 6 h) treated 293T WT and 293T FAM122A KO cells. *C*, lowering the expression of DNA-PK by KO diminishes the BLM-mediated increase of pS422-SGK1. Quantification: mean ± SD, ∗∗*p* = 0.0036 by unpaired *t* test. DMSO conditions of 293T WT and DNA-PK KO are normalized to 1. *D*, schematic of indirect (1) and direct (2) DNA-PK phosphorylation pathways of pS422-SGK1. *E*, phosphorylation level of pS422-SGK1 in cells treated with the indicated combinations of a specific inhibitor of mTORC1/2 (Torkinib, 100 nM for 30 min), inhibitor of DNA-PK (1 μM AZD7648 for 6 h), and an inducer of DNA breaks (BLM, 30 μg/ml for 6 h). *F*, lowering the expression of Rictor protein by CRISPR/Cas gene editing (KO) decreases pS473-AKT1 and pS422-SGK1 by more than 80%. *G*, cell viability of 293T transduced with lentivirus expressing mCherry, SGK1-HA, or SGK1-S422D (constitutively active) treated with ± 30 μg/ml of BLM for 2 days in the presence of a specific inhibitor of AKT (1 μM of AZD5363). Quantification: mean ± SD, ∗∗∗*p* < 0.0006 by one-way ANOVA and Dunnett’s post hoc test. *H*, viability of HeLa cells transfected with siRNA-SGK1 or scramble siRNA control treated with ± 30 μg/ml of BLM and ± 0.5 μM of AZD5363 for 5 days. Quantification mean ± SD, ∗∗*p* = 0.0073, ∗∗∗∗*p* < 0.0001 by unpaired *t* test. BLM, bleomycin; Chk1, checkpoint kinase 1; DNA-PK, DNA-dependent protein kinase; SGK1, serum- and glucocorticoid-induced kinase 1.

While exploring the effects of Chk1 on FAM122A, we noticed that several drugs that induce DNA breaks in various ways such as BLM and etoposide also increased SGK1 phosphorylation in a time- and concentration-dependent manner (Fig. 6*A*) and simultaneously increasing abundance, consistent with phosphorylation stabilizing the SGK1 protein (Fig. S3*C*).

Repair of DNA damage enables cells to function normally by a series of processes that are collectively known as the DNA damage response (DDR). As shown in Figure 6*A*, increased phosphorylation of the histone H2AX at pS139 (γH2AX) (44) and Chk1 at pS345 confirmed induction of the DDR, implying that a DDR-activated kinase leads to SGK1 phosphorylation.

The first response induced by DNA breaks is the activation of one or more of any of three related kinases: ATM (Ataxia telangiectasia mutated), ATR (ATM and Rad3-related), and DNA-dependent protein kinase (DNA-PK), each senses a different type of DNA lesions and responds activating distinct pathways to repair DNA or halt cell division (45). Their link to SGK1 was probed by inhibition of DNA-PK, which was previously identified as the kinase that mediates AKT1 phosphorylation in response to DNA damage (46, 47). The specific DNA-PK inhibitor AZD7648 suppressed BLM-induced phosphorylation of SGK1 in WT 293T and in FAM122A KO 293T cells (Fig. 6*B*). This result was confirmed in 293T cells expressing reduced levels of DNA-PK (∼20% compared to WT cells) where BLM-induced SGK1 phosphorylation was suppressed (Fig. 6*C*), meaning that SGK1 is downstream DNA-PK signaling.

As DNA-PK and mTORC2 are phosphatidylinositol-3-phosphate kinase related kinases, we considered whether SGK1 is a direct substrate of DNA-PK or alternatively, it could operate indirectly through activation of mTORC2 (Fig. 6*D*). To distinguish these possibilities, mTORC1/2 were inhibited with Torkinib (48), which does not cross-react with DNA-PK at the concentration used here (100 nM). Pharmacologic inhibition of mTORC1/2 markedly diminished pS422 levels in control and BLM-treated cells, whereas inhibition of DNA-PK (AZD7648) removed the component induced by BLM (Fig. 6*E*). To consolidate this result, we used genetic suppression of Rictor that is an essential component present in mTORC2 complex but not in mTORC1. In resting and BLM-treated RICTOR KO cells expressing ∼0.3 level of Rictor compared to WT cells, phosphorylation of pS422 in SGK1 and pS473 in AKT1 were undetectable (Fig. 6*F*), supporting DNA-PK working upstream mTORC2 as illustrated by the indirect pathway-1 of Figure 6*D*.

Finally, we examined the contribution of SGK1 to cell survival. 293T treated with BLM and with an inhibitor of AKT1 (AZD5363) to mimic cancer treatments that suppress AKT1 activity. SGK1 conferred a small (∼12%) though significant increase in 293T survival (Fig. 6*G*), whereas suppression of endogenous SGK1 in HeLa by siRNA produced the opposite effect, that is, it reduced survival of BLM-treated cells by ∼5% (Fig. 6*H*). To contextualize this finding, Figure 6*H* also shows that inhibiting AKT1 with AZD5363 leads to a decrease in viability in the presence of BLM by approximately 4%, which is comparable to that of suppressing SGK1 using siRNA. The small changes in survival imparted by SGK1 could be relevant in tumors dependent on high PI3P levels that are also treated with AKT1 inhibitors, a scenario where SGK1 would confer a competitive survival advantage and increase chances to develop resistance to chemotherapeutics.

## Discussion

A central and unanticipated result of this study is the very low phosphorylation level of SGK1 found in steady-state and growth factor–stimulated cells, implying that SGK1 is kept mostly in an inactive state. The low degree of SGK1 phosphorylation is not due to weak activity of the two upstream kinases mTORC2 and PDK1 because AKT1 exhibited robust phosphorylation in the same cells and conditions; rather, we show that it stems from relentless dephosphorylation by phosphatases, primarily PP2A and PP5. These findings implicate cellular phosphatases and their regulation as major determinants of SGK1 activity under various cellular contexts and environmental conditions. The propensity of SGK1 to undergo dephosphorylation is due to failure of the kinase domain to adopt a compact structure wherein the two crucial phosphorylated residues S422 in the HM and T256 in the activation loop are protected from access to phosphatases. Owing to structural instability, SGK1 heavily relies on protein chaperones not only for initial folding but throughout its lifetime (31). We demonstrate that SGK1 associates to the Hsp90/CDC37/PP5 chaperone complex wherein PP5 leads to SGK1 dephosphorylation in a constitutive manner and is a main driver for keeping the low phosphorylation state observed in resting cells. The function of PP5 within the chaperone complex has been acknowledged to the dephosphorylation of pS13 in CDC37 (35), an event suggested to enable the release of CDC37 from the chaperone complex; though, the exact meaning of pS13 dephosphorylation has not been yet elucidated (49). We show here that CDC37 indeed leaves the complex after being dephosphorylated by PP5 and demonstrate that the catalytic activity of PP5 extends to the client kinase within the complex, that is, SGK1, which is reset back to its original basal unphosphorylated state. Our finding broadens the role of PP5 to include dephosphorylation of kinases associated to Hsp90 in addition to previously reported steroid receptors: a set of receptors dependent on Hsp90 for refolding (50). Dephosphorylation of the glucocorticoid receptor at residues S203 and S226 by PP5 while associated to a Hsp90 chaperone complex was reported to modulate glucocorticoid receptor transcriptional activity (51).

We also found that SGK1 is a substrate of PP2A and the association of these two proteins is mediated by a few regulatory B subunits of PP2A, among which B55γ/δ also bind to FAM122A. The interaction between PP2A and FAM122A inhibits phosphatase activity, but only when S37 is phosphorylated, none of the other multiple phosphorylated sites of FAM122A impacted the phosphorylated state of SGK1. It seems from this study that the relevant kinase targeting S37 is not Chk1 as previously reported (42) because the level of pS37 did not change with specific inhibitors of Chk1 or with the activation of kinases in the upstream pathway of Chk1. Specifically, activation of the DDR by BLM did not alter pS37 levels evaluated with a specific anti-pS37-FAM122A antibody. Furthermore, none of additional agents we tested changed pS37 levels: elevation of intracellular Ca^2+^ with ATP or thapsigargin, PKA activation with forskolin, activation of AMPK with AICAR, or inhibition of cyclin dependent kinases with flavopiridol (data not shown). The molecular mechanism by which pS37-FAM122A inhibits PP2A also remains to be elucidated, though there is precedent from ENSA/Endosulfine—another endogenous inhibitor of PP2A—that turns into an inhibitor when the Greatwall kinase phosphorylates residue S67 at mitotic entry. pENSA binds much more avidly and is dephosphorylated more slowly than other substrates consequently; all other substrates retain phosphorylation for longer time and the average phosphorylation level increases (39, 52). Whether FAM122A shares a similar inhibitory mechanism remains to be elucidated.

The other significant and unexpecting contribution of this study is the demonstration that DNA damage increases SGK1 phosphorylation and that this effect is mediated by the activity of DNA-PK. Double strand DNA breaks induce autophosphorylation of DNA-PK in the nucleus where its substrates are located. Here we show that the effects of DNA-PK extend also to the cytosol where SGK1 mostly resides. This is possible because the pathway of activation of mTORC2 is indirect, that is , it may involve DNA-PK phosphorylation of mTORC2-specific subunits such as Rictor (53) or Sin1 (54) in the nuclear compartment where large fractions of these proteins are found, followed by assembly into mTORC2 complexes in the cytosolic compartment where enhanced mTORC2 activity is delivered to main substrates: SGK1.

High SGK1 expression has been reported in many types of cancer cells (16, 55), and it has been implicated in promoting cell survival and resistance to chemotherapeutic agents (17). However, abundance of transcript or protein by itself does not translate predictably into high SGK1 enzymatic activity as shown here. The significance of our findings stems from establishing the first molecular pathway directly linking DNA damage to increases in SGK1 activity and that the mechanism operates independently of growth factors and PI3K signaling. Therefore, in the context of genotoxicity, SGK1 may escape treatments targeting PI3K and AKT1 inhibition in cancer cells, a setting where the small survival advantage conferred by active SGK1 may prove most significant. Together, our main results indicate when and what conditions raise SGK1 phosphorylation from negligible levels in resting cells to many-fold higher, thereby they shed light on conditions where the impact of SGK1 activity is expected to be most significant.

## Experimental procedures

### Antibodies

Anti-SGK1 (#12103), AKT (#4691), Phospho-AKT-Ser473 (#9271), Phospho-AKT-Thr308 (#13038), Chk1(#2360), Phospho-Chk1-Ser345 (#2348), Phospho-Histone H2A.X-Ser139 (#2577), NDRG1(#9395), Phospho-NDRG1-Thr346 (#3217), Phospho-NEDD4L-Ser448 (#8063), Hsp90 (#4877), CDC37(#4793), HA Tag (#3724), and FLAG Tag (#14793) were from Cell Signaling Technology. Phospho-SGK1-S422 (#44-1264G), FAM122A (#MA5-24510), V5-HRP (#R961-25) were from Invitrogen; NEDD4-2 (#ab131167) from Abcam; PP5 antibody (#sc-271816) from Santa Cruz Biotechnology; FAM122A (#NBP2-31646) from Novus Biologicals; GAPDH-HRP (#BE0034-100) from Easybio; actin-HRP (#AM8502b) from ABGENT; and secondary antibodies, anti-rabbit IgG HRP (#A16110), and anti-mouse IgG HRP (#A16072) from Invitrogen.

### Plasmids

Constructs were generated by PCR of the corresponding coding DNA (cDNA) with tags -HA, FLAG, V5, or Strep and cloned into pCDNA3.1 vector using Q5 high-fidelity DNA polymerases (#M0491). Site-directed mutations were introduced with Agilent PfuTurbo DNA Polymerase (#600257).

### Generation of phospho-antibodies

All procedures related to animals were approved by Tsinghua Institutional Animal Care and Use Committee (Animal Protocol No.17-CC2). SGK1 pS422 and pT256 antibodies were made to synthetic phosphopeptides corresponding to mouse SGK1 amino acid residues 416-429 (EAFLGFpSYAPPVDSC) and residues 249-262 (EHNGTTSpTFCGTPEC) with an additional cysteine in the N terminus. These peptides were conjugated to keyhole limpet hemocyanin (mcKLH) (#77666, Thermo Fisher Scientific) and used as antigens for immunization of New Zealand White rabbits. Antibodies recognizing nonphospho-SGK1 were subtracted from immunized sera by passing through corresponding unphosphorylated peptide (same sequence as above without phosphorylation) chromatographic columns. Phospho-antibodies were further affinity purified from the subtracted fraction on phosphopeptide chromatographic columns (#20401; Thermo Fisher Scientific). ELISA and western blots were performed to assess the antibody specificity. Lamda PP (#P0753S, NEB) was used to dephosphorylate SGK1 proteins according to manufacturer’s instructions. FAM122A phospho-S37 antibody was generated by immunizing rabbits with synthetic phosphopeptides corresponding to human FAM122A amino acid residues 34-40 (RSNpSAPL) (Dia-An Biotechnology).

### Cell culture

HEK293T (CL-0005) from Procell, HeLa (CCL-2), A549 (CCL-185), MCF7 (HTB-22) from ATCC, MDA-MB-231 (gift from Dr Hanqiu Zheng, Tsinghua University), KPC2 (gift from Dr Charles David, Tsinghua University) were cultured at 37 °C in a humidified 5% CO_2_ incubator in high glucose Dulbecco’s modified Eagle’s medium (HyClone) supplemented with 10% fetal bovine serum (BI), 1% penicillin, and 1% streptomycin (Hyclone).

### Generation of stable cell lines

FAM122A, PP5, or DNA-PK KO 293T cells were generated by CRISPR/Cas9 system. sgRNA targeting FAM122A sequence: 5′-GCAGATAAGCCACTCCTGGG-3′ as previously described (42), sgRNA targeting PP5 sequence: 5′-ACGCGCTGGGAGACGCCACG-3′, sgRNA targeting DNA-PK sequence: 5′-GTGTGCGTTGCTCCCTGCTG-3′. Single clones were selected with 1 μg/ml puromycin. RICTOR KO 293 cells were purchased from Ubigene. SGK1-HA, SGK1-S422D-HA, and mCherry stably overexpressed 293T cells were generated by infection of lentivirus packaged in 293T.

### Transfection

Cells were transfected with plasmid DNAs or siRNAs using Lipofectamine 2000 according to manufacturer’s directions. SiRNAs targeting SGK1 were designed as previously described (56).

### Drug treatments

Serum starvation was carried out in Dulbecco’s modified Eagle’s medium for 20 h. Drugs used were as follows unless otherwise indicated: insulin (20 μg/ml; 30 min; #12585-014, Invitrogen), insulin like growth factor 1 (250 ng/ml; 30 min; #PMG0075, Invitrogen), torin 1 (200 nM; 30 min; #S2827, Selleck), torkinib (100 nM; 30 min; #HY-10474, MedChemExpress), pictilisib (1 μM; 2 h; #11600, Cayman Chemical), rapamycin (20 ng/ml; 30 min; #HY-10219, MedChemExpress), Calyculin A (100 nM; 15 min; # 9902, Cell Signaling Technology), okadaic acid (1 μM; 1 h; #5934, Cell Signaling Technology), tautomycetin (1 μM; 1 h; #B7009, APEBIO), BLM (30 μg/ml; 6 h; #S1214, Selleck), etoposide (1 μM; 24 h; #S1225, Selleck), prexasertib (100 nM; 6 h; #S6385, Selleck), AZD7648 (1 μM; 6 h; #S8843, Selleck), AZD5363 (10 μM; 4 h; #S8019, Selleck).

### Cell lysis and immunoprecipitation

Cells were washed twice with cold DPBS (#CC010, Macgene) and then lysed with TENT 1% (50 mM Tris, 150 mM NaCl, 5 mM EDTA, 1% Triton X-100, pH 7.4) on ice in the presence of protease inhibitors (#4693132001, Roche) and phosphatase inhibitors cocktail (#04906837001, Roche). Cell lysates were centrifuged at 13, 500*g* for 5 min at 4 °C to remove debris. Protein concentrations were determined with the BCA Protein Assay kit (#PC0020, Solarbio). Equal amounts of total protein from lysates were immunoprecipitated with FLAG magnetic beads (#M8823, Sigma-Aldrich), HA magnetic beads (#88837, Pierce), or rabbit polyclonal antibody with Protein G agarose (#20398, Pierce) and then eluted with SDS-PAGE loading buffer. Co-immunoprecipitations of PP5 and SGK1 were carried out with Strep-Tactin XT Superflow (#2-4010-010, Neuromics) and eluted with buffer BXT (0.1 M Tris–Cl, 0.15 M NaCl, 1 mM EDTA, 50 mM Biotin (#6-6325-001, IBA), pH 8.0) or with HA magnetic beads.

### Western blots

Equal amounts of whole cell lysate proteins or immunoprecipitants were loaded to 12% SDS-PAGE (Bio-Rad, #1610175) and transferred to polyvinylidene difluoride membranes (#BSP0161, PALL). Membranes were blocked with 5% skim milk in TBS-T (2 mM Tris, 13.7 mM NaCl, 0.01% Tween 20, pH 7.6) and then probed with indicated primary and secondary antibodies. Chemiluminescence was detected with Millipore Immobilon Western HRP Substrate (#WBKLS0500) in Bio-Rad ChemiDocTM XRS+.

### Analysis of FAM122A phosphorylation in cells

293T cells transfected with FAM122A-HA were treated with 30 μg/ml BLM or dimethylsulfoxide for 6 h and then lysed with TENT 1% supplemented with protease and phosphatase inhibitors. FAM122A was immunoprecipitated with HA magnetic beads and resolved on 12% SDS⁄PAGE. Bands containing FAM122A were visualized with ImperialTM Protein Stain (#24615, Thermo Fisher Scientific) and excised for trypsin digestion, followed by LC-MS/MS analysis (Thermo-Dionex Ultimate 3000 HPLC system interfaced to a Thermo Scientific Q Exactive mass spectrometer). The Q Exactive mass spectrometer was operated using Xcalibur 2.2 software (ThermoFisher: Xcalibur Software Catalog number: OPTON-30965), and the MS/MS spectra were searched against human FAM122A from UniProt using Proteome Discoverer (Version PD1.4, Thermo-Fisher Scientific). Site phosphorylation ratio of FAM122A was calculated by the peak area of phosphorylated peptides *versus* total peptides corresponding to that site.

### Quantitative RT-PCR analysis

Total RNA was extracted from cells using TRIzol Reagent (#15596018, Invitrogen), and single strand cDNA was prepared by reverse transcription of total RNA using ProtoScript II First Strand cDNA Synthesis Kit (#E6560S, NEB). Quantitative RT-PCR was carried out using RealStar Green Fast Mixture with ROX (#A303-05, GenStar) in StepOnePlus Real Time PCR System (Applied Biosystems). Primer sequences were as follows: SGK1, F5′-GCAGAAGAAGTGTTCTATGCAGT-3′ and R5′-CCGCTCCGACATAATATGCTT-3′; GAPDH, F5′- ACAACTTTGGTATCGTGGAAGG-3′ and R5′- GCCATCACGCCACAGTTTC-3′.

### Cell viability assays

293T cells were infected with lentivirus packaged in 293T cells. After at least 10 days of selection with 1 μg/ml puromycin, cells were seeded in 96-well plates at a concentration of 1000 cells per well. Twenty four hours later, cells were treated with 1 μM AZD5363 and 30 μg/ml BLM for 2 days. HeLa cells transfected with scramble or SGK1 targeting siRNA were seeded at a concentration of 2000 cells per well and treated with 30 μg/ml BLM for 5 days 24 h post transfection. Cell viability was assessed using Cell-Titer Glo (#G7570, Promega) according to manufacturer’s instructions.

### Statistical analysis

Western blot signals were quantified using Image J software (https://imagej.nih.gov/ij/). Statistical analyses and visualizations were performed by GraphPad Prism 9 (Graphpad.com). All data are represented as mean ± SD, and significance was tested using unpaired Student’s *t* test or one-way ANOVA as indicated.

## Data availability

All data supporting the findings of this study are in the manuscript.

## Supporting information

This article contains supporting information.

## Conflict of interest

The authors declare that they have no conflicts of interest with the contents of this article.

## Supporting information

KinaseFinder Results

FAM122A PTM-MS
